# Mucormycosis in the COVID-19 Environment: A Multifaceted Complication

**DOI:** 10.3389/fcimb.2022.937481

**Published:** 2022-07-18

**Authors:** Rohit Sharma, Praveen Kumar, Abdur Rauf, Ashun Chaudhary, Pradeep Kumar Prajapati, Talha Bin Emran, Clara Mariana Gonçalves Lima, Carlos Adam Conte-Junior

**Affiliations:** ^1^ Department of Rasa shastra and Bhaishajya Kalpana, Faculty of Ayurveda, Institute of Medical Sciences, Banaras Hindu University, Varanasi, UP, India; ^2^ Department of Medicinal Chemistry, Faculty of Ayurveda, Institute of Medical Sciences, Banaras Hindu University, Varanasi, UP, India; ^3^ Department of Chemistry, University of Swabi, Swabi, Pakistan; ^4^ Department of Plant Science (Botany), Central University of Himachal Pradesh, Dharamshala, India; ^5^ Department of Rasashastra and Bhaishajya Kalpana, All India Institute of Ayurveda, New Delhi, India; ^6^ Department of Pharmacy, BGC Trust University Bangladesh, Chittagong, Bangladesh; ^7^ Department of Pharmacy, Faculty of Allied Health Sciences, Daffodil International University, Dhaka, Bangladesh; ^8^ Department of Food Science, Federal University of Lavras, Lavras, MG, Brazil; ^9^ Center for Food Analysis (NAL), Technological Development Support Laboratory (LADETEC), Federal University of Rio de Janeiro (UFRJ), Cidade Universitária, Rio de Janeiro, Brazil

**Keywords:** mucormycosis, SARS-CoV-2, diabetes, steroids, amphotericin-B, COVID-19, GRP78, hepcidin

## Abstract

The second wave of coronavirus disease 2019 (COVID-19) caused severe infections with high mortality. An increase in the cases of COVID-19-associated mucormycosis (CAM) was reported predominantly in India. Commonly present in immunocompromised individuals, mucormycosis is often a life-threatening condition. Confounding factors and molecular mechanisms associated with CAM are still not well understood, and there is a need for careful research in this direction. In this review, a brief account of the diagnosis, management, and advancement in drug discovery for mucormycosis has been provided. Here, we summarize major factors that dictate the occurrence of mucormycosis in COVID-19 patients through the analysis of published literature and case reports. Major predisposing factors to mucormycosis appear to be uncontrolled diabetes, steroid therapy, and certain cancers. At the molecular level, increased levels of iron in COVID-19 might contribute to mucormycosis. We have also discussed the potential role and regulation of iron metabolism in COVID-19 patients in establishing fungal growth. Other factors including diabetes prevalence and fungal spore burden in India as contributing factors have also been discussed.

**Graphical Abstract:**
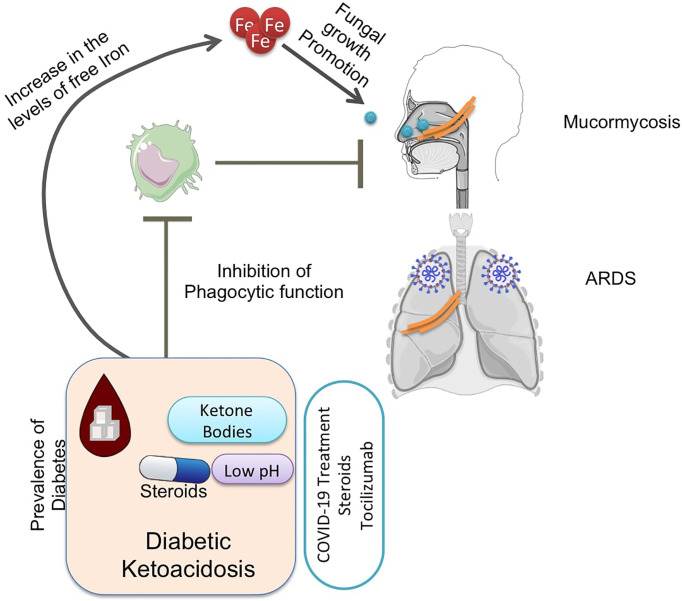


## Highlights

1. Mucormycosis infections were observed mostly in India during the second wave of the coronavirus disease 2019 (COVID-19), which was associated with a very high mortality and morbidity.
2. Mucorales iron metabolism plays a crucial role in the establishment of infection, which might be attributed to severe acute respiratory syndrome coronavirus 2 (SARS-CoV-2) infection and comorbidities.
3. Host iron metabolism regulation by hepcidin might be an important factor in determining the availability of iron to fungi during the infection.
4. Diabetic ketoacidosis is a major predisposing comorbid condition that can help Mucorale infection through upregulation of glucose-regulated protein 78 (GRP78), elevating levels of free iron and impairing the immune response.
5. Steroid therapy and related immunosuppressant therapies dampen the immune response and build a ground for mucormycosis establishment.
6. The Indian population exhibits a high prevalence of diabetes, and fungal spore burden is also high in India.
7. Amphotericin B is the most effective drug against mucormycosis. However, there is a need to develop new drugs to fight this deadly infection.

## 1 Introduction

Even after more than 2 years of its discovery, the coronavirus disease 2019 (COVID-19) is storming the world with rapid infections and has been declared a pandemic by the World Health Organization (WHO). This pandemic has resulted in 531,550,610 infections resulting in 6,302,982 deaths as of June 9, 2022 (WHO, 2021). Severe acute respiratory syndrome coronavirus 2 (SARS-CoV2) infection results in upper respiratory illnesses, collectively referred to as COVID-19. Ranging from asymptomatic to flu-like mild symptoms to acute respiratory distress syndrome (ARDS) (C.S.G. of the I.C. @ on T. @ of V, 2020), COVID-19 has a variety of symptoms in patients and most of the patients recover on their own. However, in patients with ARDS, it is usually fatal. Host immune response is activated upon infection, and hyperactivation of the immune system is believed to cause damage to the lung tissues (Tang et al., 2020). A lung biopsy from a COVID-19 patient showed alveolar damage and increased activation of CD4+ and CD8+ lymphocytes (Xu et al., 2020; Tian et al., 2020).

While secondary infections during COVID-19 are commonly reported, a surge in mucormycosis infections in COVID-19 patients was surprising during the second wave across the world, especially in India. Mucormycosis cases were reported in the highest number among COVID-19 patients (Singh et al., 2021), along with other fungal infections like aspergillosis (White et al., 2020). While mucormycosis is not a new infection, it is resulting in a huge fatality and morbidity in patients with SARS-CoV-2 infection. The complete pathophysiology of the complication is not yet understood. Therefore, a detailed investigation into the possible causes and treatment options in COVID-19-associated mucormycosis (CAM) is needed.

Several factors might affect host immunity that could be instrumental in understanding the occurrence of CAM. COVID-19-mediated tissue damage and inflammation might increase the levels of free iron in the serum and cause a concomitant increase in serum ferritin levels (Cheng et al., 2020). Iron metabolism is vital for mucorale fungi and might contribute to the development of mucormycosis post-COVID-19. Apart from this, diabetic ketoacidosis (DKA) is one of the major contributing factors to mucormycosis where low pH and hyperglycemia play a vital role in fungal angioinvasion. Glucocorticoid usage for ARDS treatment during COVID-19 dampens the immune response and also might induce hyperglycemia, and it, therefore, is considered one of the prominent causes. Apart from these, certain cancers, organ transplants, certain supplements, and environmental factors such as personal hygiene have been speculated as potential contributors to CAM. However, a precise understanding of the contributing factors and mechanisms behind CAM is still lacking.

In this review, we are summarizing the diagnosis and treatment and potential contributing factors and detailing the potential molecular mechanisms that lead to the establishment of the CAM. Understanding the mechanisms associated with CAM will help to reduce the cases of mucormycosis post-COVID-19, which is vital for drug discovery.

## 2 The Danger of COVID-19-Associated Mucormycosis

Mucormycosis and other fungal infections are caused by fungal species, which are normally present in the environment. Despite being rare compared to bacterial infections, the mortality associated with fungal infections is very high (Brown et al., 2012; Garbee et al., 2017). Certain diseases or medical conditions, which predispose individuals to an immunocompromised state, make patients susceptible to mucormycosis. Various species that cause mucormycosis (Rhizopus oryzae, Mucor circinelloides, Rhizomucor pusillus, Saksenaea vasiformis, Rhizopus microsporus, Apophysomyces variabilis, Lichtheimia ramosa, Cunninghamella bertholletiae) belong to the order Mucorales of the kingdom Fungi. Patients with organ transplants, cancers such as leukemia, HIV infection, health conditions requiring chronic steroid treatments, usage of immunosuppressive drugs, and most notably uncontrolled diabetes with ketoacidosis, fall in the risk group for mucormycosis and other fungal infections (Lewis and Kontoyiannis, 2013; Binder et al., 2014). Additionally, Candida sp. and Aspergillus sp. are also known to cause significant infections in immunocompromised individuals (Thornton, 2020).

Several Rhizopus sp. are known to cause the most frequent infections, although aspergillosis is also a commonly encountered infection in the risk group of COVID-19 patients. Inhalation of fungal spores present in the air is the major route of infection, although infection through ingestion and skin contact can also occur. Rhino-orbito-cerebral mucormycosis (ROCM) is the most prevalent form of mucormycosis with a very high mortality even with the proper medications. Various mechanisms that contribute to the increased fungal infections include factors such as extensive angioinvasion, increased fungal virulence, and a delay in the diagnosis of the fungal infections (Katragkou et al., 2014). Neutrophils are the blood cells implicated in the host defense against fungal infections. Neutrophils migrate toward fungal pathogens and mediate fungal killing through phagocytosis, oxidative stress, and neutrophil extracellular traps (NETs) (Urban and Nett, 2019). Neutrophil functions such as migration, oxidative burst, and NET formation are modified in diabetic subjects, leading to decreased clearance of fungal pathogens (Dowey et al., 2021). Certain fungi such as Rhizopus can metabolize ketone bodies present in the patients with DKA resulting in angioinvasion, thrombosis, and ischemic tissue necrosis (Katragkou et al., 2014; Lin et al., 2017). Patients with pulmonary mucormycosis develop symptoms akin to pneumonia, which can also spread to the heart (Katragkou et al., 2014; Lin et al., 2017; Sahota et al., 2017; Thornton, 2020). Mucorale infections have immensely contributed to COVID-19-related ROCM, leading to high rates of mortality and morbidity (Fouad et al., 2021).

## 3 Diagnosis and Management of COVID-19-Associated Mucormycosis

Most of the CAM infections result from inhalation of fungal spores or from direct inoculation into the skin at the site of injury or inside the intestinal mucosa like typical fungal infections (Chakrabarti and Singh, 2014). Microscopic examination of the biopsy, culture studies, and advanced imaging technologies are used to detect fungal infections. The diagnosis for mucormycosis is typically made by analyzing the histology of the tissue samples from biopsy samples and confirmed by the presence of hyphae in the sample (Hernández and Buckley, 2021).

ROCM remains the most common presentation of CAM along with pulmonary, cutaneous, and disseminated types. In India, ROCM is associated with diabetes mellitus (Chakrabarti and Singh, 2014), and the hematological malignancies are associated with pulmonary infections (Klimko et al., 2014).

Mucormycosis is a very aggressive fungal infection caused by a variety of Mucorales fungi. The European Society for Clinical Microbiology and Infectious Diseases and the European Confederation of Medical Mycology Joint Clinical Guidelines recommend direct diagnosis of mucormycosis using microscopy with optical brightness, histopathology, and culture. Imaging studies are recommended to determine the extent of the spread of infection (Cornely et al., 2014).

Amphotericin B is the preferred drug for the treatment of mucormycosis with simultaneous management of the underlying cause such as blood sugar and surgical debridement of the infected and necrotic tissue (Long and Koyfman, 2015; Skiada et al., 2018). Administration of antifungal therapy at the earliest and usage of adjunct therapies should follow for effective mucormycosis management (Cornely et al., 2014; Tissot et al., 2017). For patients with uncontrolled blood sugar, usage of sodium bicarbonate and insulin administration help to reverse ketoacidosis (Gebremariam et al., 2016). Corticosteroids and other immunosuppressive therapies should be reduced to the minimum quickly. Early diagnosis followed by treatment with antifungals significantly improves the mortality associated with mucormycosis (Chamilos et al., 2008; Gebremariam et al., 2016). Isavuconazole is the only new drug for mucormycosis. However, it does not provide superior effects compared to amphotericin B or posaconazole (Skiada et al., 2018).

Amphotericin B exhibits limited efficacy against some species of *Cunninghamella* and *Apophysomyces* (Almyroudis et al., 2007; Alvarez et al., 2010). Posaconazole, isavuconazole, and itraconazole are additional drugs that exhibit activity against Mucorales fungi. The recommended dosage of these drugs varies between 5–10 mg/kg/day (Cornely et al., 2014; Tissot et al., 2017). Isavuconazole is a newly discovered agent with a broad-spectrum antifungal activity. Amphotericin B in combination with caspofungin or posaconazole was also suggested as a treatment option. This combination was not effective in hematological patients but was effective for ROCM (Kyvernitakis et al., 2016). An iron chelator, deferasirox, has also been implicated in the treatment of mucormycosis as an adjunct therapy (Ibrahim et al., 2007). However, more research is needed to evaluate its benefits and side effects. Hyperbaric oxygen along with administration of certain cytokines (Roilides et al., 2014) is another adjunct therapy that is expected to enhance the host immune response against the fungi. Surgical debridement should be very aggressive and healthy tissue surrounding the necrotic tissues should also be removed, as the spread of infection could be very high in the tissues. Surgical options are better for ROCM infections and also for the removal of a single pulmonary lesion. Plastic surgery could be performed where required (Skiada et al., 2018).

### 3.1 Advancements in Drug Discovery for Mucormycosis

VT-1161 is an inhibitor of fungal CYP51, which has activity against mucorales fungi. Additional studies are needed before it could be prescribed to patients to treat mucormycosis (Wiederhold et al., 2018). Another promising antifungal agent, APX001A, targets Gwt1, which is involved in the posttranslational modifications of surface proteins in eukaryotic cells. APX001A has entered phase 2 clinical trials (clinical trial identifier NCT04240886). Usage of additional antifungal agents (e.g., itraconazole and terbinafine) was found effective in a single-center clinical trial (Gupta et al., 2022), which requires further investigation. Other potential antifungal drugs such as SCH 42427 (Goldani and Sugar, 1994), colistin (Ben-Ami et al., 2010), and PC1244 (Colley et al., 2018) also exhibit potency against mucormycosis.

## 4 Association of Mucormycosis with SARS-CoV-2 Infections: Case Studies

We gathered published data on CAM patients until July 9, 2021, from PubMed using the keywords, “COVID-19 and Mucormycosis” in order to gain insight into the contributing factors for CAM. The data consisted of case studies where individual patient information was available according to parameters of our study design. While a few reports about CAM have emerged after we collected the data, our analysis truly presents the extent of the confounding factors of CAM. **Table 1** summarizes the information on CAM according to country.

**Table 1:** Worldwide reported cases of COVID-19-associated mucormycosis.

Country	N	MeanAge, years	Gender	Comorbidity	Treatment	Infection Site	Outcome	References (PMID)
**Austria**	1	53	M = 1	Leukemia = 1	Steroids = 1	Pulmonary = 1	Dead = 1	33513875
**Brazil**	1	86	M = 1	None = 1	None = 1	Gastrointestinal = 1	Dead = 1	33207116
**Egypt**	20	52.15	F = 9, M = 11	Diabetes = 17, Leukemia = 2, Renal disease = 1	None = 20	ROCM = 20	Alive = 11, Dead = 9	34087330, 34124087
**France**	1	55	M = 1	Lymphoma = 1	None = 1	Pulmonary = 1	Dead =1	33527098
**India**	68	51.52	F = 15, M = 53	Diabetes = 49, None = 12, Hypothyroidism = 1, Renal transplant = 2, Coronary artery disease = 1, Hypertension = 2, Rheumatoid arthritis = 1	Steroids = 54, None = 14	Gastrointestinal = 2, Osteomyelitis = 2, Pulmonary = 2, ROCM = 62	None = 2, Alive = 48, Dead = 18	33145132, 33463566, 33544266, 33716414, 33727483, 33903850, 33964720, 34011758, 34026593, 34052046, 34081817, 34128074, 34167998, 34177157, 34178609, 34181023, 34215642
**Iran**	19	51.63	F = 8, M = 11	Diabetes = 15, Hematological malignancy = 2, Hypertension = 1, None = 1	Steroids = 11, None = 8	ROCM = 19	Alive = 11, Dead = 8	33713565, 34096653, 34237015, 33843287
**Italy**	1	66	M = 1	Hypertension = 1	None = 1	Pulmonary = 1	Dead = 1	33331988
**Mexico**	1	24	F = 1	Diabetes = 1	None = 1	None = 1	Dead = 1	33575155
**Netherlands**	4	60	M = 4	Diabetes = 2, None = 2	Steroids = 4	ROCM = 1, Pulmonary = 3	Alive = 1, Dead = 3	34114540
**Spain**	2	55	M = 2	Diabetes, Renal disease = 1, Renal disease = 1	Steroids = 2	ROCM = 1, Musculoskeletal = 1	Alive = 2	34038014
**Turkey**	11	51.72	F = 9, M = 2	Diabetes = 8, Hypertension = 2, Myelodysplastic syndrome = 1	Steroids = 11	ROCM	Alive = 4, Dead = 7	34057620
**UK**	2	22	M = 2	None = 2	Steroids = 1, None = 1	Pulmonary = 2	Dead = 2	34075329, 32844161
**USA**	10	55.6	F = 1, M = 9	None = 3, Heart transplant = 1, Diabetes = 6	Steroids = 7, None = 2, Vaccine = 1	Pulmonary = 3, Cutaneous = 1, Multiple/Thoracic = 1, ROCM = 5	Alive = 4, Dead = 6	33670842, 33752571, 33752571, 33857916, 32983308, 33842203, 33984095, 33229953, 34222572, 32972795

ROCM, rhino-orbito-cerebral mucormycosis.

The possibility of fungal infections in COVID-19 was speculated in the early stages of the pandemic (Song et al., 2020; Gangneux et al., 2020). The second wave of COVID-19 has indeed witnessed a huge rise in mucormycosis infections. However, other fungal infections have also been reported (White et al., 2020). Apart from the studies in the **Table 1**, some other reports such as 287 cases from India (Patel et al., 2021), 23 cases from India (Sharma et al., 2021), and another 21 cases (Ravani et al., 2021) were not included because of the lack of individual patient profile according to our study design. In a study, authors found a difference in the bacterial and fungal species that cause secondary infections in the social setting and in patients with a prolonged hospital admission (Westblade et al., 2021). Another recent report summarized mucormycosis cases (n = 101) where majority (n = 82) of the cases were reported from India (Singh et al., 2021). A very comprehensive recent study among the Indian population described 2,826 cases of rhino-orbito-cerebral cases of mucormycosis (Sen et al., 2021).

Several countries such as the United States, United Kingdom, Turkey, Iran, Egypt, Netherlands, and India have reported cases of CAM. However, majority of cases were found in India (**Table 1** and Raut and Huy (2021)).

**Figure 1:**
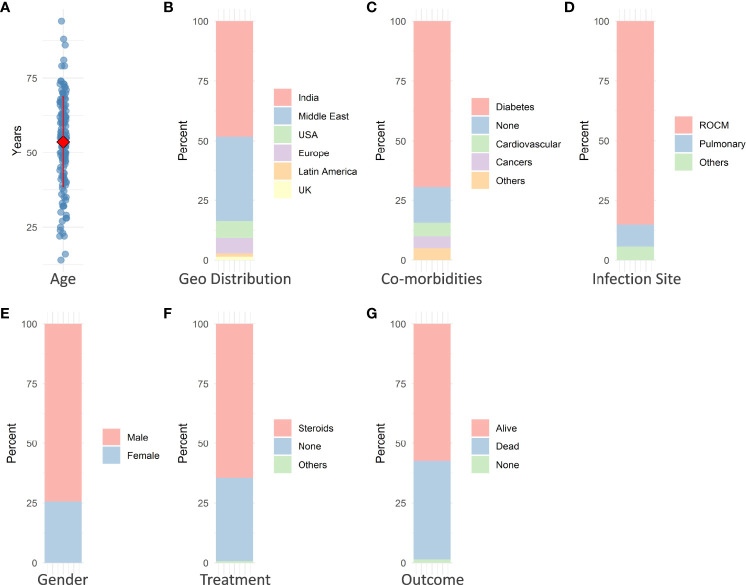
Some of the major observable factors in COVID-19-associated mucormycosis. **(A)** Mean age of patients is ~54 years. **(B)** Most of the cases were reported from India (>48%). **(C)** Majority of the patients were diabetic (~70%). **(D)** Rhino-orbito-cerebral mucormycosis (ROCM) predominated in occurrence (~85%). **(E)** Men (~75% of total patients) were infected more than women. **(F)** Approximately 65% of patients underwent steroid therapy. **(G)** More than 41% of mucormycosis infections resulted in patient death.

The mean age of patients with CAM is ~54 years (**Figure 1A**), suggesting middle age as a risk factor. The immune system with advancing age gets impaired in its ability to fight infections (Weyand and Goronzy, 2016). Among several comorbidities such as diabetes, cancer, hypertension, renal disease, and transplants, diabetic patients were presented with most of the infections (**Figure 1C**). DKA predisposes patients toward mucormycosis through multiple pathways.

The most prevalent type of infection is ROCM in COVID-19 patients (**Figure 1D**). Interestingly, many features of CAM such as ROCM and hyperglycemia are also found in diabetic patients, indicating a common mechanism between the two conditions. Men seem (75% of cases) to be more prone to mucormycosis compared to women with COVID-19 (**Figure 1E**). The percentage of mucormycosis infections where patients were treated with steroids was higher (**Figure 1F**). Steroids are well known to decrease immunity, and further chronic steroid usage develops into glucocorticoid-induced diabetes mellitus (GIDM) (Buchman, 2001; Calvet and Yoshikawa, 2001). The occurrence of infection might also depend on environmental conditions with an abundance of certain fungal species. Mucormycosis infections dominated the occurrence of infections in our analysis (**Figure 1F**). CAM is highly lethal, and about 41% of deaths were reported from the CAM (**Figure 1G**).

**Figure 2:**
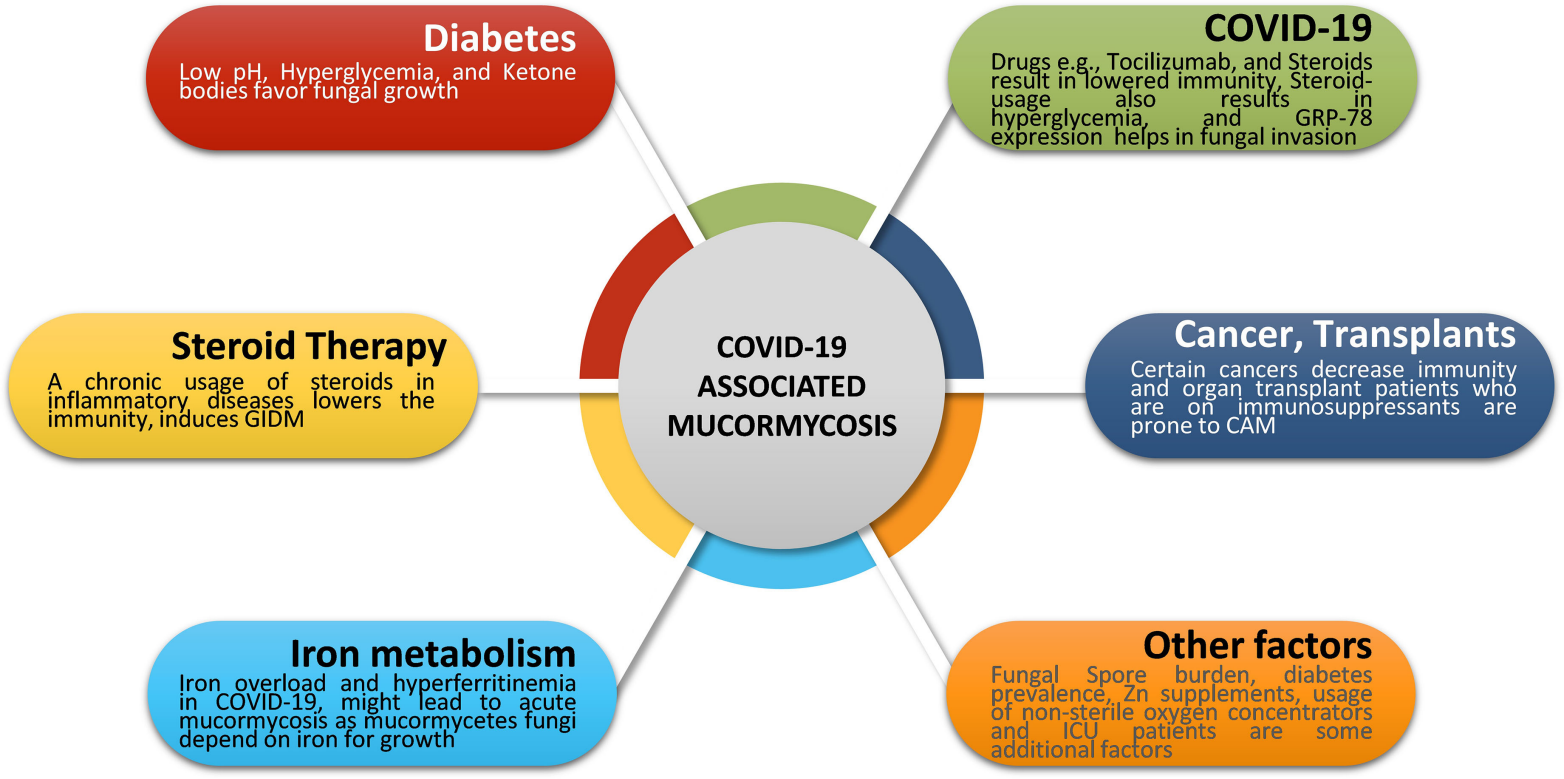
Major risk factors in CAM. Fungal infections typically occur due to a compromised immune status. Several factors such as diabetes, iron metabolism, COVID-19 treatments, steroid therapy, organ transplants, and certain cancers represent the major risk factors for mucormycosis. Apart from these, fungal spore burden, prevalence of diabetes, zinc supplements, and hospital environment might also contribute to mucormycosis cases post-COVID-19. COVID-19, Coronavirus disease 2019; GRP-78, glucose-regulated protein 78; CAM, COVID-19 associated mucormycosis; GIDM, Glucocorticoid-Induced Diabetes Mellitus; ICU, Intensive Care Unit.

Some cases of mucormycosis were also present in certain cancer patients with COVID-19 infections. Combined together, the presentation of cases in India is much higher compared to those in other countries (**Figure 1B**). **Figure 2** denotes some of the major contributing factors that contribute to establishing mucormycosis in COVID-19 patients.

## 5 Molecular Mechanisms in Establishing COVID-19-Associated Mucormycosis

Several factors such as iron metabolism, expression of glucose-regulated protein 78 (GRP78), and neutropenia are speculated to play a role in establishing CAM in COVID-19 patients.

### 5.1 GRP78 Expression, SARS-CoV-2 Infection, and Mucormycosis

Apart from angiotensin-converting enzyme-2 (ACE-2), the GRP78 receptor on endothelial cells plays an important role in COVID-19. A molecular chaperone, GRP78 acts as a coreceptor along with ACE-2 (Prakash et al., 2021). Elevated serum levels of GRP78 were reported from SARS-CoV-2-infected patients (Sabirli et al., 2021). Blocking the GRP78 receptor might reduce viral internalization to host cells.

**Figure 3:**
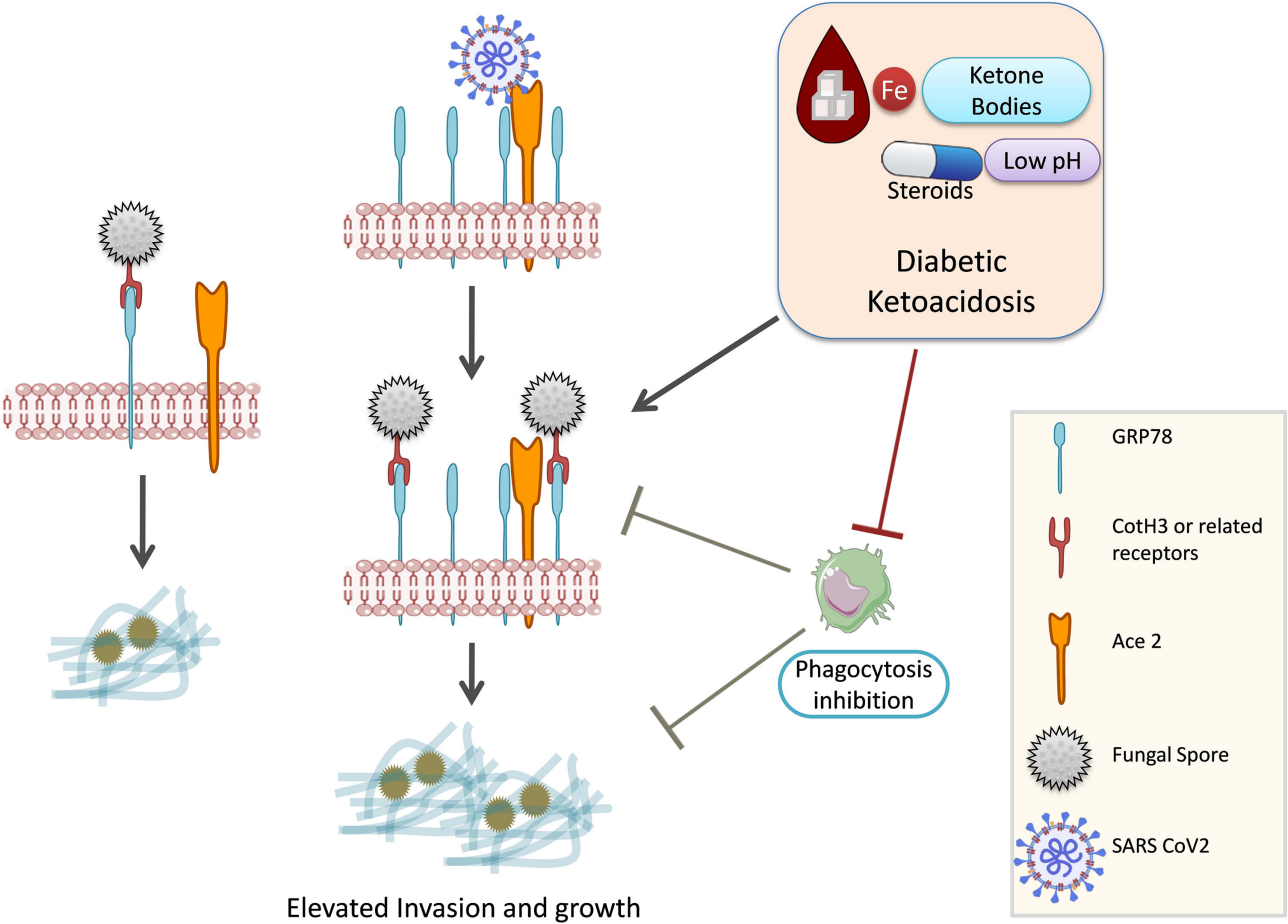
GRP78, COVID-19, and mucormycosis. Mucorale fungi utilize GRP78 receptor for host cell invasion. SARS-CoV-2 infection increases the expression of GRP78 and hence might promote mucormycosis. Diabetic ketoacidosis and steroid usage are also known to upregulate GRP78 expression. GRP-78, glucose-regulated protein 78; SARS CoV-2, Severe acute respiratory syndrome coronavirus 2; Ace 2, Angiotensin-converting enzyme 2; CotH3, spore coat protein homolog 3.

Spore coat protein (CotH3) of *Rhizopus arrhizus* utilizes endothelial GRP78 as a receptor (Gebremariam et al., 2019; Alqarihi et al., 2020). *Rhizopus delemar* interaction with GRP78 on nasal epithelial cells leads to invasion and damage of the nasal epithelium (Alqarihi et al., 2020). The expression of both CotH3 and GRP78 is enhanced by high glucose, iron, and ketone body levels, which are hallmark features of DKA (**Figure 3**). Therefore, GRP78 might be a positive molecular link between COVID-19 and mucormycosis (Prakash et al., 2021). In contrast, *R. delemar* CotH7 recognizes integrin β1 as a receptor on alveolar epithelial cells, causing the activation of epidermal growth factor receptor (EGFR) and leading to host cell invasion (Alqarihi et al., 2020). Pulmonary fungal invasion is carried out through CotH7 protein interaction with integrin beta and EGFR activation. EGFR inhibitor treatment (cetuximab) increased the survival of mice with pulmonary mucormycosis (Alqarihi et al., 2020). The high-affinity iron permease is a key virulence factor required for *R. oryzae* pathogenesis (Ibrahim et al., 2010). Bicarbonate correction of ketoacidosis alters host–pathogen interactions and alleviates mucormycosis (Gebremariam et al., 2016). Anti-CotH3 antibodies protect mice from mucormycosis by preventing invasion and augmenting opsonophagocytosis (Gebremariam et al., 2019).

### 5.2 Neutropenia

Neutrophils are critical for fighting mucormycosis, and therefore, neutropenia is a risk factor (Ibrahim et al., 2012). While the association of neutropenia with COVID-19 is not well established, diabetes is typically accompanied by neutropenia (Alba-Loureiro et al., 2007). Additionally, functions of neutrophils including phagocytosis, migration, and intracellular reactive oxygen species (ROS) generation are impaired in hyperglycemic conditions (Dowey et al., 2021). The modulation of neutrophil functions in DKA remains a subject of investigation.

### 5.3 Iron Metabolism, COVID-19, and Mucormycosis

**Figure 4:**
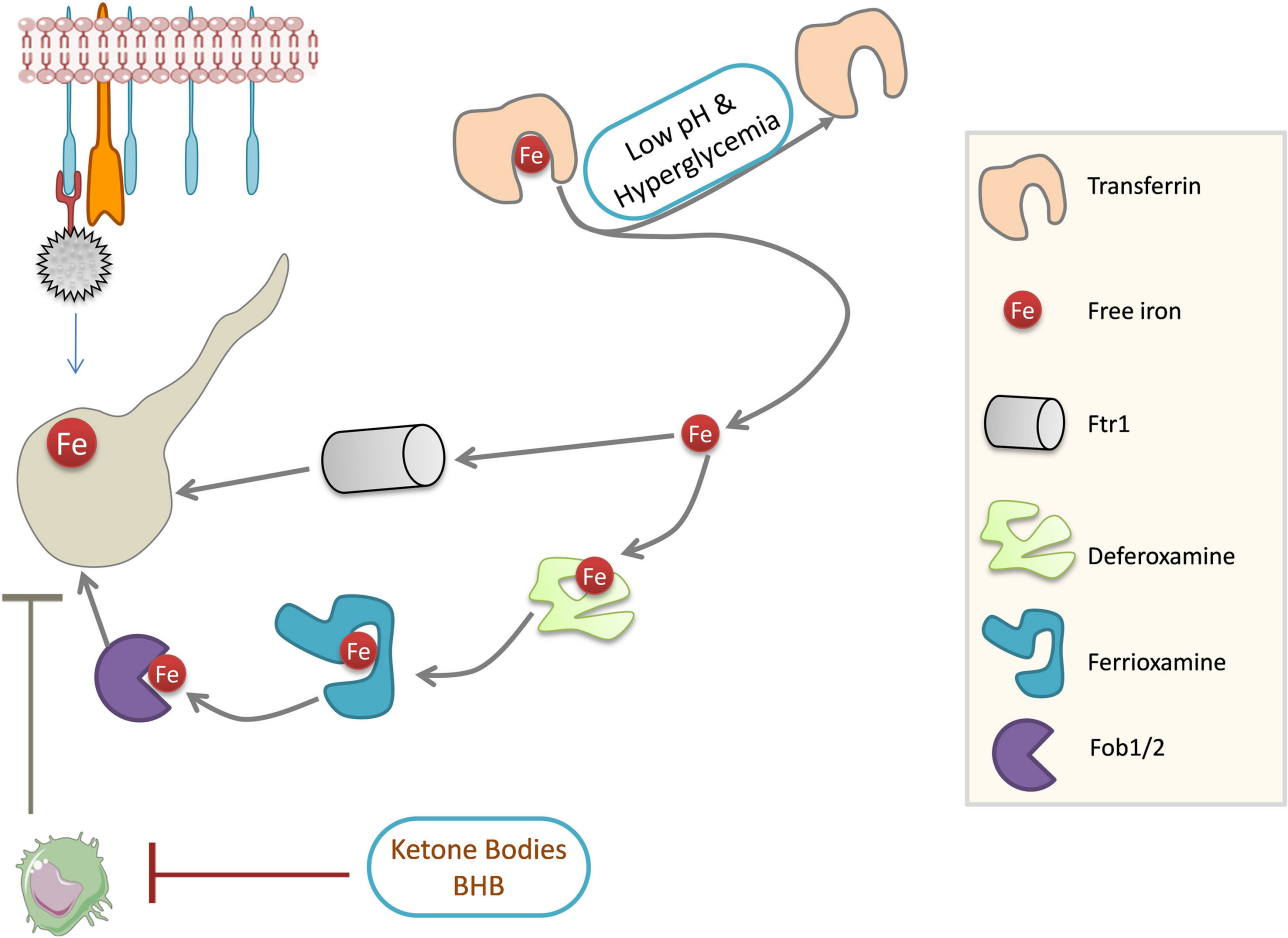
Mucorales iron metabolism. Iron is required for Mucorale growth. Fungi can obtain iron through permease Ftr1 and Fob1/2 system. Strong iron affinity molecules can obtain iron from the iron chelator, deferoxamine, which is ultimately acquired by fungi for growth. Iron-binding activity of transferrin is impaired in DKA patients: low pH dissociates iron from transferrin and high glucose concentrations can glycosylate transferrin, resulting in the release of free iron. Ketone bodies impair phagocytosis and dampen the immune response in DKA patients. Ftr1: high-affinity iron permease 1 (Fe TRansporter); Fob1/2, ferrioxamine binding (Fob) proteins 1/2; BHB, Beta-Hydroxybutyrate; DKA, Diabetic ketoacidosis.

Iron is a crucial nutrient whose concentration is limited by its binding to the iron-sequestering proteins such as transferrin, lactoferrin, and ferritin. Accumulation of free iron is prevented for two main reasons: iron is involved in ROS production, which is detrimental to cells, and free iron is critical for the growth of certain pathogens such as mucorales. Iron-binding proteins act as host defense against the mucorales (Artis et al., 1982). DKA patients with low pH have elevated free iron in the serum and support the growth of mucorales (Ibrahim et al., 2007). However, serum without free iron does not support the growth of mucorales (Ibrahim, 2011), suggesting the importance of free iron in the establishment of mucormycosis. An increase in iron is favorable for the growth of mucorale fungi, which possess an extensive network of iron acquisition genes (**Figure 4**). These include high-affinity iron permease (FTR1), ferric reductase, and multicopper oxidase (Prakash et al., 2017). Mucorales can store iron in the form of ferritins (Ibrahim et al., 2008), and siderophore transporters and heme oxygenases also help in iron acquisition by the fungus (Prakash et al., 2017). Disruption of FTR1 function in the DKA mouse model reduced fungal virulence (Ibrahim et al., 2010), which shows the significance of iron metabolism in mucorale virulence. Iron chelation using deferasirox (Ibrahim et al., 2007) and deferiprone (Ibrahim et al., 2006) decreased fungal load in the tissues of DKA mice and improved the survival rate, as siderophores in the fungi cannot utilize iron from these proteins unlike another iron chelator deferoxamine, which increased fungal virulence and pathogenesis (Daly et al., 1989; Boelaert et al., 1991; Boelaert et al., 1993).

SARS-CoV-2 attacks hemoglobin, causing dissociation of porphyrins, leading to free iron in the circulation (Liu and Li, 2022). Iron in free form (Fe^2+^) catalyzes the production of ROS and leads to inflammation through various pathways (Banchini, 2020) and tissue damage (Dixon and Stockwell, 2014), including heavy damage to the lungs in COVID-19 infection (Habib et al., 2021). Hyperferritinemia is associated with COVID-19 infections (Cheng et al., 2020). Iron sequestration by ferritin is a host defense mechanism to deny access of iron to pathogens (Cheng et al., 2020) such as mucorales, which rely highly on serum iron for growth pathogenesis. Hyperferritinemia is positively correlated with inflammation and is frequently associated with high mortality (Kernan and Carcillo, 2017). Free iron also causes inflammation through ferroptosis and pulmonary injury and promotes thrombosis during COVID-19 (Habib et al., 2021). The iron chelator lactoferrin, having a higher affinity than that of transferrin, might be a potential therapy to reduce free iron from the bloodstream. Lactoferrin expression increases upon viral entry and can limit SARS-CoV-2 cell entry by hindering the binding of the virus with surface heparin sulfate proteoglycans (Habib et al., 2021). Therefore, it is plausible to use a suitable iron chelator to treat patients with mucormycosis.

### 5.4 Regulation of Iron Stores by Hepcidin

**Figure 5:**
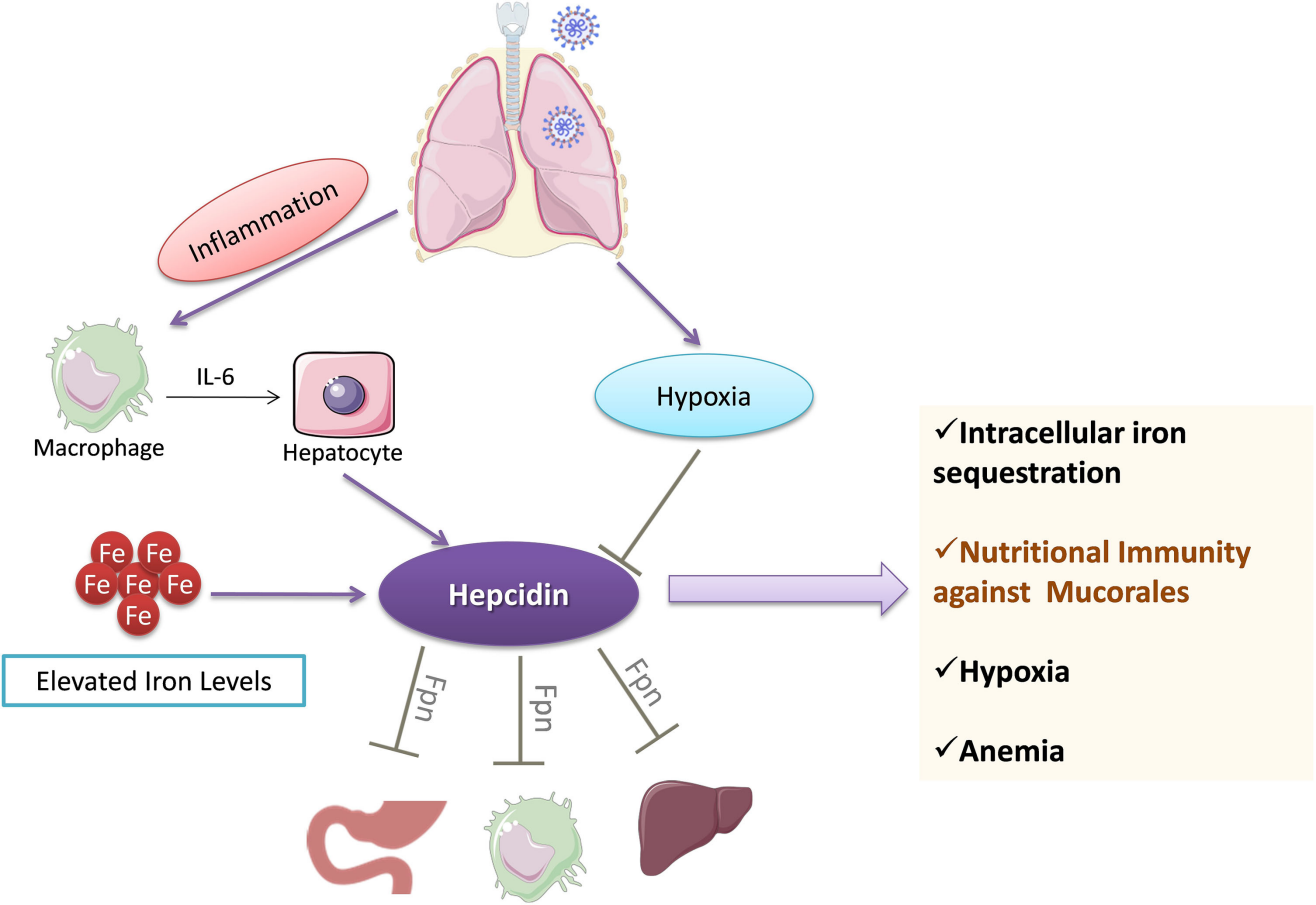
Regulation of iron stores by hepcidin during COVID-19. Hepcidin levels are increased following IL-6-mediated action on hepatocytes. Hepcidin sequesters iron levels inside the cells by downregulating ferroportin (Fpn). The decrease in iron levels leads to anemia and ultimately culminates in hypoxemia. Hepcidin-mediated decrease in iron levels may provide protection against pathogens such as mucorales fungi. IL-6, Interleukin-6; Fpn, Ferroportin.

Hepcidin is a hormone secreted by the liver, which controls the levels of iron. Ferroportin is an iron transporter, and hepcidin binding to ferroportin leads to internalization of the complex, thus preventing iron transport (Nemeth and Ganz, 2009). COVID-19 is associated with increased levels of hepcidin and ferritin (**Figure 5**), reflecting a load of free iron on the patients (Nai et al., 2021). Hyperferritinemia is a hallmark of inflammation and correlates with poor patient prognosis. A high iron burden is one of the major predisposing factors for mucormycosis.

## 6 Mucormycosis: Is it Really a COVID-19-Related Problem?

COVID-19 and its treatments alter the host immune system, making the patient prone to secondary infections. There is no significant evidence to suggest that SARS-CoV-2 infection directly leads to CAM. The only relationship could be the free iron release from the tissue damage as a consequence of COVID-19. An *in silico* study concluded that SARS-CoV-2 might attack host hemoglobin during infection (Liu and Li, 2022). However, more studies are required to substantiate this notion.

### 6.1 Environmental and Epidemiological Factors

#### 6.1.1 Fungal Spore Burden

Fungal infections are typically spread by spores. The spore burden is quite high in developing countries, especially in India (Richardson and Rautemaa-Richardson, 2019). Several pathogenic *Mucor* species (*R. arrhizus*) including rare mucorale species (*A. variabilis* and *Rhizopus homothallicus*) have been isolated in India (Richardson and Rautemaa-Richardson, 2019; Prakash et al., 2016).

#### 6.1.2 Prevalence of Diabetes

The burden of diabetes in India is very high (Tandon et al., 2018). Hyperglycemia and steroid therapy interfere with phagocytic function, and thereby, the clearance of the fungal spores and their germination are not checked, providing a suitable platform for fungal growth (**Figures 3**, **4**).

#### 6.1.3 Other Factors

The risk of mucormycosis among COVID-19 patients treated in an intensive care unit (ICU) could be significantly higher than that in non-COVID-19 patients (Seidel et al., 2022). Apart from this, the sterility of water used in oxygen concentrators was also suggested as a contributing factor (Kumar et al., 2021). However, in a recent study, the requirement of oxygen concentrators and hospital admission did not affect the occurrence of CAM (Arora et al., 2022). Nasal mucosa is a delicate structure, and high-temperature steam could harm the membrane, which could be a suitable ground for fungal invasion (Kumar et al., 2021). This however needs further validation.

### 6.2 Role of Mucormycosis-Predisposing Comorbidities

#### 6.2.1 Diabetic Ketoacidosis

Hyperglycemia results in excessive glycosylation of proteins including transferrin and ferritin (59). This results in the release of free iron from these iron storage proteins. DKA in which blood pH is lowered by ketone bodies further impairs the iron chelation ability of transferrin. A consequent surge in ketone bodies, iron, and glucose promotes fungal growth (Baldin and Ibrahim, 2017). These three also upregulate the expression of GRP78 on endothelial cells and increased the expression of spore coat protein CotH3 (**Figure 3**). Binding of CotH3 with GRP78 results in angioinvasion and epithelial damage (Baldin and Ibrahim, 2017) and is crucial for establishing mucormycosis. In addition to fungal growth promotion, ketone bodies, glucose, and unbound iron suppress host immunity (Puchalska and Crawford, 2017). Hyperglycemia also contributes to altered iron metabolism, causing hyperferritinemia (Prakash et al., 2021). Hyperglycemia can result from either underlying causes such as diabetes or steroid therapy (Hwang and Weiss, 2014), damage to the pancreatic beta cells by SARS-CoV-2 infection (Hwang and Weiss, 2014), and stress-related elevation in the levels of cortisol (Dias et al., 2020; Prakash et al., 2021).

#### 6.2.2 Role of COVID-19 Treatments

COVID-19 treatments for ARDS predominantly include the usage of glucocorticoids for immunosuppression (Singh et al., 2020). While steroids make COVID-19 less severe, prolonged steroid usage suppresses the host immune system, which might lead to an increased risk of mucorale infection (Ahmadikia et al., 2021). Additionally, chronic steroid usage might induce hyperglycemia-like conditions resulting in ketoacidosis (Hwang and Weiss, 2014). DKA is a known risk factor for mucormycosis.

Corticosteroid usage along with others was a major factor that resulted in various invasive filamentous fungal infections in intensive care unit (ICU) settings (Bassetti and Bouza, 2017). In addition to being immunosuppressive and promoting infections, corticosteroid usage has been reported to induce diabetes-like conditions by inducing hyperglycemia (Tamez-Pérez et al., 2015; Suh and Park, 2017). Ketone bodies in hyperglycemic conditions can be utilized by fungi for growth, and a low pH in hyperglycemic patients further favors fungal growth (**Figures 3**, **4**) (Katragkou et al., 2014; Lin et al., 2017).

Azithromycin, an antibiotic given in COVID-19 treatment to prevent secondary infections, is known to inhibit IL-6 (Bouwman et al., 2004), which is crucial for antimicrobial defense. Furthermore, in a related clinical study, usage of azithromycin was not recommended during SARS-CoV-2 infection (Butler et al., 2021). Other immunosuppressant drugs such as tocilizumab dampen the immune response against new infections. Among Indian populations, unsupervised usage of antibiotics and other drugs is quite common (Thakar and Lal, 2021), which also might be a contributing factor to CAM.

Zinc supplementation as part of COVID-19 management has been controversial. In a study, various fungal species in mucorales were subjected to zinc deprivation, which inhibited fungal growth (Staats et al., 2013; Leonardelli et al., 2019). Zinc chelators along with amphotericin B and posaconazole were effective against 6 *Mucor* species (Leonardelli et al., 2019). A study found zinc supplementation to be protective in CAM (Arora et al., 2022). Nonetheless, more studies are required to conclude whether zinc supplementation confers protection or makes individuals susceptible to mucormycosis.

#### 6.2.3 Influence of Host Microbiome in COVID-19

Several studies have shown differential microbiome composition in COVID-19 patients (Dhar and Mohanty, 2020; Wu et al., 2021; Yamamoto et al., 2021; Hussain et al., 2021). The altered composition of microbiome and mycobiome might explain the severity and prevalence of mucormycosis post-COVID-19. Additionally, establishing a correlation between diabetic patients and mucormycosis will further help understand how microbiome plays a contributing factor. A detailed investigation is warranted in this direction.

## 7 Conclusions and Future Perspectives

Elevated inflammation in response to SARS-CoV-2 infections leads to cytokine storms resulting in ARDS and severe lung damage. Corticosteroid usage in order to curb the inflammation 1) lowers the immune response and 2) induces GIDM on prolonged usage. Both of these conditions are very suitable for infections in particular fungal growth. DKA is a predisposing factor to mucormycosis. Patients with uncontrolled diabetes are characterized by a rapid development of mucormycosis. Since iron metabolism is vital for mucorale growth, the release of iron in patients following tissue damage from SARS-CoV-2 infection might be one of the major factors that determines the establishment of mucorale infection. The mucorale spore burden and prevalence of diabetes are very high in India. Both of these factors probably contributed profusely in the appearance of a number of CAM cases in India. Apart from these, other factors such as sterility of water in oxygen concentrators and differential presence of pathogens in the hospitals have been suggested. These remain a subject of further investigation.

Treating mucormycosis in later stages is extremely difficult, and liposomal amphotericin B remains the first line of treatment. Therapies combining amphotericin B and iron chelators could be more effective. A combination of iron chelators with antifungal medications should be tested against Mucorale fungal infections in controlled clinical trials. Since complications of mucormycosis expand into various dimensions, a multifaceted team composed of experts from infectious diseases, hematology, endocrinology, microbiology, nursing, and pharmacy can result in optimal patient health. Although there is already some development in the field of novel agents (VT-1161) for mucormycosis management, there is a desperate need for rapid development of additional antifungal agents.

## Author Contributions

Conceptualization, data collection and writing-original manuscript, RS; Editing and proofreading, PK, AR, TE, AC, PP, CG, and CC-J. All authors approved submission of the final manuscript.

## Conflict of Interest

The authors declare that the research was conducted in the absence of any commercial or financial relationships that could be construed as a potential conflict of interest.

## Publisher’s Note

All claims expressed in this article are solely those of the authors and do not necessarily represent those of their affiliated organizations, or those of the publisher, the editors and the reviewers. Any product that may be evaluated in this article, or claim that may be made by its manufacturer, is not guaranteed or endorsed by the publisher.
